# Resveratrol Promotes Wound Healing by Enhancing Angiogenesis via Inhibition of Ferroptosis

**DOI:** 10.1002/fsn3.70254

**Published:** 2025-05-05

**Authors:** Yujie Pan, Mingyan Xia, Jin Luo, Shuai Lu

**Affiliations:** ^1^ Department of Traumatic Orthopedics The Affiliated Hospital of Guizhou Medical University Guiyang Guizhou China; ^2^ School of Clinical Medicine, Guizhou Medical University Guiyang Guizhou China; ^3^ Department of Anatomy School of Basic Medicine Science, Guizhou Medical University Guiyang China; ^4^ Department of Biology School of Basic Medical Science, Guizhou Medical University Guiyang China

**Keywords:** angiogenesis, diabetic wound healing, ferroptosis, resveratrol

## Abstract

Diabetic wound healing critically depends on functional endothelial cells for angiogenesis, yet the hyperglycemic microenvironment induces endothelial dysfunction through oxidative stress, inflammation, and senescence. Although ferroptosis has been recognized as a critical pathological factor contributing to impaired diabetic wound healing, the therapeutic potential of resveratrol (Res), a natural polyphenol with well‐documented antioxidant and anti‐ferroptotic properties, remains underexplored in this context. This study aimed to investigate the protective effects of Res on endothelial cells and elucidate its underlying mechanisms in diabetic wound healing. In vitro experiments systematically evaluated Res's impact on cellular inflammatory responses, senescence levels, and angiogenic capacity. Subsequent in vivo studies assessed Res's therapeutic potential by monitoring diabetic wound healing progression and analyzing associated histological changes. To clarify the mechanisms underlying Res's promotion of diabetic wound healing, we conducted comprehensive analyses measuring intracellular reactive oxygen species, lipid peroxidation levels, mitochondrial membrane potential and morphology, ferroptosis‐related marker expression, and upstream signaling pathway regulation. Res significantly reduced HG‐induced inflammatory responses and cellular senescence in human umbilical vein endothelial cells while enhancing their angiogenic potential in vitro. In vivo results showed that Res not only markedly accelerated diabetic wound healing but also demonstrated multiple beneficial effects, including effective suppression of cellular senescence, decreased ferroptosis levels, and significantly promoted angiogenesis. Mechanistic investigations confirmed that Res achieves these effects by inhibiting ferroptosis through activation of the PI3K‐AKT‐Nrf2 signaling axis. Our results demonstrate that Res protects endothelial cells from HG‐induced ferroptosis by activating PI3K‐AKT‐Nrf2 signaling, thereby promoting angiogenesis and diabetic wound healing. These findings highlight Res as a promising therapeutic candidate for impaired diabetic wound repair and justify further clinical investigation.

## Graphical abstract

Resveratrol attenuates intracellular inflammatory responses and cellular senescence by inhibiting ferroptosis via activating the PI3K‐AKT‐Nrf2 signaling pathway, ultimately facilitating angiogenesis and healing of diabetic wounds.

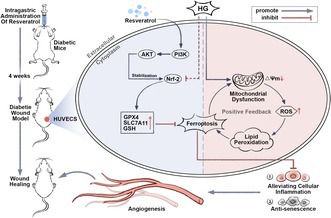

**Abbreviations**
AGEs: advanced glycation end products
AKT: phosphorylation of protein kinase B
AOD: average optical density
BCA: bicinchoninic acid
BSA: bovine serum albumin
CCK‐8: cell counting kit‐8
ELISA: enzyme‐linked immunosorbent assay
FBS: fetal bovine serum
GSH: glutathione
GSSG: oxidized glutathione
HG: high glucose
HMGB1: high mobility group box 1 protein
IL‐1β: interleukin 1 beta
MDA: malondialdehyde
MMP: mitochondrial membrane potential
Nrf2: nuclear factor erythroid 2‐related factor 2
PI3K: phosphoinositide 3‐kinase
PVDF: polyvinylidene difluoride
Res: resveratrol
RIPA: radioimmunoprecipitation assay
ROS: reactive oxygen species
SA‐β‐Gal: senescence‐associated β‐galactosidase
SDS‐PAGE: sodium dodecyl sulfate‐–polyacrylamide gel electrophoresis
STZ: streptozotocin
TNF‐α: tumor necrosis factor alpha

## 1 Introduction

Diabetes mellitus (DM) is a pervasive chronic metabolic disorder with a global prevalence. According to recent data published in *The Lancet*, it is projected that approximately 828 million individuals globally will be affected by diabetes by 2022 (NCD Risk Factor Collaboration (NCD‐RisC) 2024). The majority of significant complications associated with diabetes are related to microvascular damage, including diabetic retinopathy, diabetic nephropathy, neuropathy, and impaired wound healing. It is estimated that between one‐third and one‐fifth of individuals with diabetes will experience chronic nonhealing wounds at some point in their lives (Chang and Nguyen 2021). Despite the substantial medical resources that have been invested, existing treatments are not fully effective in preventing the risk of amputation or restoring full skin function (Dixon and Edmonds 2021). Therefore, there is an urgent need to develop new drugs for the treatment of diabetic wound healing.

Distinct from other chronic wounds, diabetic wounds with delayed healing exhibit more complex and unique microenvironmental characteristics, including elevated local glucose levels and an accumulation of advanced glycation end products (AGEs). These factors collectively impede the healing process of diabetic wounds (Patel et al. 2019; Shao et al. 2023). Prolonged exposure to elevated glucose concentrations can inhibit the proliferation and impair the function of fibroblasts and keratinocytes, thereby adversely affecting the healing of diabetic wounds (Singh et al. 2023). Additionally, the hyperglycemic microenvironment induces oxidative stress and inflammation in endothelial cells, leading to endothelial dysfunction and dysangiogenesis (Liu, Zhang, et al. 2022). In turn, impaired angiogenesis exacerbates the local wound microenvironment, further impairs the angiogenesis process, and ultimately impedes wound healing (Xiong et al. 2023). Numerous studies indicate that persistently elevated local glucose levels can also induce ferroptosis through the reactive oxygen species (ROS)‐mediated depletion of glutathione peroxidase and lipid peroxidation (Icli et al. 2019). There is an overactivation of ferroptosis in delayed healing wounds associated with diabetes, and inhibiting ferroptosis can facilitate wound healing (Cui et al. 2023).

Resveratrol (Res) is a non‐flavonoid polyphenol compound found in a variety of plants and exhibits various biological activities, such as anti‐aging, anti‐inflammatory, antioxidative stress, and immune regulation (Cong et al. 2021; Shahwan et al. 2022). It has been widely used in the research of cardiovascular diseases and neurodegenerative diseases (Ungurianu et al. 2023; Yu et al. 2013). In recent years, Res has garnered significant attention in the field of diabetes wound treatment due to its excellent antioxidant and anti‐inflammatory properties (Li et al. 2024; Zhou et al. 2021). The mechanism by which Res affects endothelial cell injury in a high‐glucose environment remains under‐researched. Consequently, this study examined the protective effects of Res on human umbilical vein endothelial cells (HUVECs) damaged by high glucose (HG) conditions through both in vivo and in vitro experiments. Additionally, we conducted preliminary investigations into its underlying mechanisms, with the aim of identifying potential therapeutic strategies to enhance the healing of diabetic wounds.

## 2 Materials and Methods

### 2.1 Chemicals and Regents

Res was purchased from Aladdin Reagents (Shanghai, China). Antibodies against IL‐1β (#82696‐15‐RR, 1:2000), HMGB1 (#66525‐1‐Ig, 1:2000), TNF‐α (#60291‐1‐Ig,1:1000), P21 (#10355‐1‐AP, 1:1000), P16 (#10883‐1‐AP, 1:1000), CD31 (#28083‐1‐AP, 1:5000), GPX4 (#67763‐1‐Ig, 1:1000), and SLC7A11(#26864‐1‐AP, 1:1000) were obtained from Proteintech (Wuhan, Hubei, China). The antibody against Nrf2 (#A21176, 1:2000) was purchased from ABclonal (Wuhan, Hubei, China). Antibodies against PI3K (#EM1701‐62, 1:1000), AKT (#ET1609‐47, 1:2000), and p‐AKT (#ET1607‐73, 1:2000) were purchased from HUABIO (Hangzhou, Zhejiang, China). The Lipid Peroxidation MDA Assay Kit, Senescence β‐Galactosidase Staining Kit, GSH and GSSG Assay Kit, ROS Assay Kit, and Mitochondrial Membrane Potential Assay Kit with JC‐1 were obtained from Beyotime (Haimen, Jiangsu, China). The Cell Counting Kit‐8 was acquired from Dojindo Laboratories (Kumamoto, Japan). ELISA Kits for IL‐1β and TNF‐α were sourced from Lianke (Hangzhou, Zhejiang, China).

### 2.2 Cell Culture

HUVECs were obtained from ZQXZBIO (Shanghai, China) and cultured in RPMI 1640 Medium (Gibco, USA) supplemented with 10% fetal bovine serum (FBS; Gibco, USA). The cells were incubated in a humidified atmosphere at 37°C with 5% CO_2_. Prior to any experimental treatments, the cells were allowed a 24‐h acclimation period. Detailed descriptions of the drug treatments are provided in the figure legends.

### 2.3 Cell Counting Kit‐8 Assay

The Cell Counting Kit‐8 (CCK‐8) assay was employed to assess relative cell viability (Wang et al. 2022). Briefly, cells were seeded at a density of 5 × 10^3^ cells per well in a 96‐well plate and cultured for 24 h. After a specific intervention, the culture medium was replaced, and 10 μL of the CCK‐8 working solution was added to each well. The plate was incubated in the dark for 2 h. Following this, the absorbance at 450 nm was measured using a spectrophotometer.

### 2.4 Western Blot

Skin tissues or cells were harvested and subjected to lysis using a radioimmunoprecipitation assay (RIPA) buffer, supplemented with 1% protease and phosphatase inhibitor. The lysis process was conducted at 4°C for 20 min, with intermittent ultrasonic disruption. Subsequently, the lysate was collected by centrifugation at 14,000 rpm for 20 min at 4°C. Protein concentration was determined using a bicinchoninic acid (BCA) Protein Assay Kit (Thermo Scientific, USA). Approximately 10 μg of protein was resolved by sodium dodecyl sulfate–polyacrylamide gel electrophoresis (SDS‐PAGE) and then transferred onto a polyvinylidene difluoride (PVDF) membrane. The membrane was subsequently blocked with 5% skim milk at ambient temperature for a duration of 2 h. Following this blocking step, it was incubated overnight at 4°C with the specific primary antibody, and afterward, it was incubated at room temperature for 2 h with the corresponding secondary antibody. The protein bands were ultimately visualized using a chemiluminescence detection method and analyzed with ImageJ software.

### 2.5 Enzyme Linked Immunosorbent Assay

The levels of IL‐1β and TNF‐α in HUVECs were assessed using an enzyme‐linked immunosorbent assay (ELISA). The culture medium from each group was collected and centrifuged at 3000 rpm for 10 min at 4°C. The concentrations of IL‐1β and TNF‐α were then quantified according to the manufacturer's protocol for the ELISA kit.

### 2.6 Senescence‐Associated β‐Galactosidase Staining Assay

To assess the anti‐senescence effects of Res on HG‐treated HUVECs, senescence‐associated β‐galactosidase (SA‐β‐Gal) staining was performed (Gonzalez‐Gualda et al. 2020). A commercially available SA‐β‐Gal Staining Kit was used according to the manufacturer's protocol. Senescent cells were identified by the presence of blue staining. The percentage of positively stained cells was determined by quantifying the number of stained cells in five randomly selected microscopic fields.

### 2.7 Tube Formation Assay In Vitro

HUVECs with different interventions were collected and seeded at a density of 1.5 × 10^4^ cells per well on a 96‐well plate pre‐coated with Matrigel (BD Biocoat, USA). After incubation for 6 h, the tubes were stained with Calcein‐AM and then observed with a fluorescence microscope. AngioTool v.2 software was used to quantitatively evaluate the degree of tube formation.

### 2.8 Diabetic Models

The establishment of the diabetic mouse model referred to previous experiments (Han et al. 2021). Following acclimatization to the environment, 4‐week‐old male C57BL/6J mice were randomly assigned to the normal control group (NC), the diabetic group (DM), and the Res treatment group (Res). Both the DM and Res groups were placed on a high‐sugar, high‐fat diet, with weekly monitoring of body weight and blood glucose levels. At 12 weeks of age, the DM and Res groups were administered a single intraperitoneal injection of streptozotocin (STZ) at a dose of 40 mg/kg (Sigma‐Aldrich, St. Louis, MO, USA) to induce the destruction of pancreatic beta cells. Mice exhibiting blood glucose concentrations exceeding 16.7 mmol/L were subsequently diagnosed with diabetes. During the experiment, the mice were provided with a standard diet under controlled environmental conditions, maintained at 25°C and 55% relative humidity.

### 2.9 Wound Healing Assessment In Vivo

Following the successful induction of DM, the Res group received an intragastric administration of 50 mg/kg per day for a duration of 4 weeks (Bi, Qin, et al. 2023). Subsequently, standardized full‐thickness skin wounds, each with a diameter of 10 mm, were created in all groups. Briefly, under abdominal anesthesia using 1% pentobarbital sodium, the dorsal skin of the subjects was shaved and disinfected. A scalpel was then used to create full‐thickness skin wounds. Wound assessments and photographic documentation were conducted on days 4, 7, and 14. On day 14, the subjects were euthanized. The wound area and healing rate were quantified using ImageJ software.

### 2.10 Hematoxylin and Eosin Staining

Skin tissue samples were fixed in 4% paraformaldehyde, embedded in paraffin, and sectioned. Wound healing was assessed using hematoxylin and eosin (H&E) staining according to established protocols. The width of newly formed epithelial tissue was quantified with ImageJ software.

### 2.11 Immunohistochemistry

Paraffin‐embedded tissue sections underwent standard dewaxing procedures, followed by antigen retrieval via thermal treatment with sodium citrate. To reduce non‐specific staining, endogenous peroxidase activity was inhibited using 3% hydrogen peroxide, and nonspecific binding sites were blocked with 5% bovine serum albumin (BSA). The specific primary antibody was then applied, and the sections were incubated at 4°C overnight. Subsequently, the secondary antibody was administered and incubated in the dark at room temperature for 1 h. Nuclear staining was performed using DAPI. The sections were then subjected to DAB staining solution for immunohistochemical analysis, followed by subsequent microscopic imaging.

### 2.12 Intracellular Glutathione Detection

In accordance with the established protocol, the treated HUVECs were harvested via centrifugation. Subsequently, a protein removal buffer was added to the cell pellet, followed by two rapid freeze–thaw cycles and cell lysis using liquid nitrogen and a water bath maintained at 37°C. The resultant lysate was centrifuged at 12,000 rpm for 10 min at 4°C. The supernatant obtained from this process was utilized for the quantification of glutathione (GSH) and oxidized glutathione (GSSG) levels (Yuan et al. 2021).

### 2.13 Intracellular Malondialdehyde Detection

Malondialdehyde (MDA) is an endogenous byproduct of lipid peroxidation, and its concentration serves as a biomarker for assessing the extent of lipid oxidative degradation (Jiang et al. 2023). The Lipid Peroxidation MDA Assay Kit is employed to analyze cellular samples post‐intervention, following the manufacturer's protocol.

### 2.14 Reactive Oxygen Species Assay

DCFH‐DA permeates the cell membrane and undergoes oxidation by reactive oxygen species (ROS) to yield the fluorescent compound DCF, thereby allowing the fluorescence intensity to serve as an indicator of intracellular ROS levels (Ding, Sun, et al. 2022). HUVECs, pretreated with or without HG and Res, were cultured in a 6‐well plate for 24 h. Subsequently, an appropriate concentration of DCFH‐DA was introduced into the plate and incubated in the dark at 37°C for 20 min. The fluorescence intensity was then quantified using fluorescence spectroscopy.

### 2.15 Membrane Potential Assay and Mitochondrial Morphology

To evaluate the mitochondrial membrane potential (MMP), the JC‐1 assay was employed (Yang et al. 2024). After JC‐1 staining, HUVECs from different treatment groups were analyzed using flow cytometry, adhering to the manufacturer's protocol. For mitochondrial morphology observation, HUVECs were initially fixed with 3% glutaraldehyde and subsequently post‐fixed using 1% osmium tetroxide. Following fixation, the cells underwent a dehydration process using a graded series of acetone concentrations and were then embedded in Epon 812 resin. Ultrathin sections were stained with uranyl acetate and lead citrate for enhanced contrast and examined by transmission electron microscopy.

### 2.16 Statistical Analysis

Statistical analyses were conducted utilizing GraphPad Prism 9. The data are presented as the mean ± standard deviation. The statistical significance was assessed using one‐way analysis of variance (ANOVA), with a *p*‐value of < 0.05 considered indicative of significance.

## 3 Results

### 3.1 Res Alleviates HG‐Induced Cellular Inflammation in HUVECs

**FIGURE 1:**
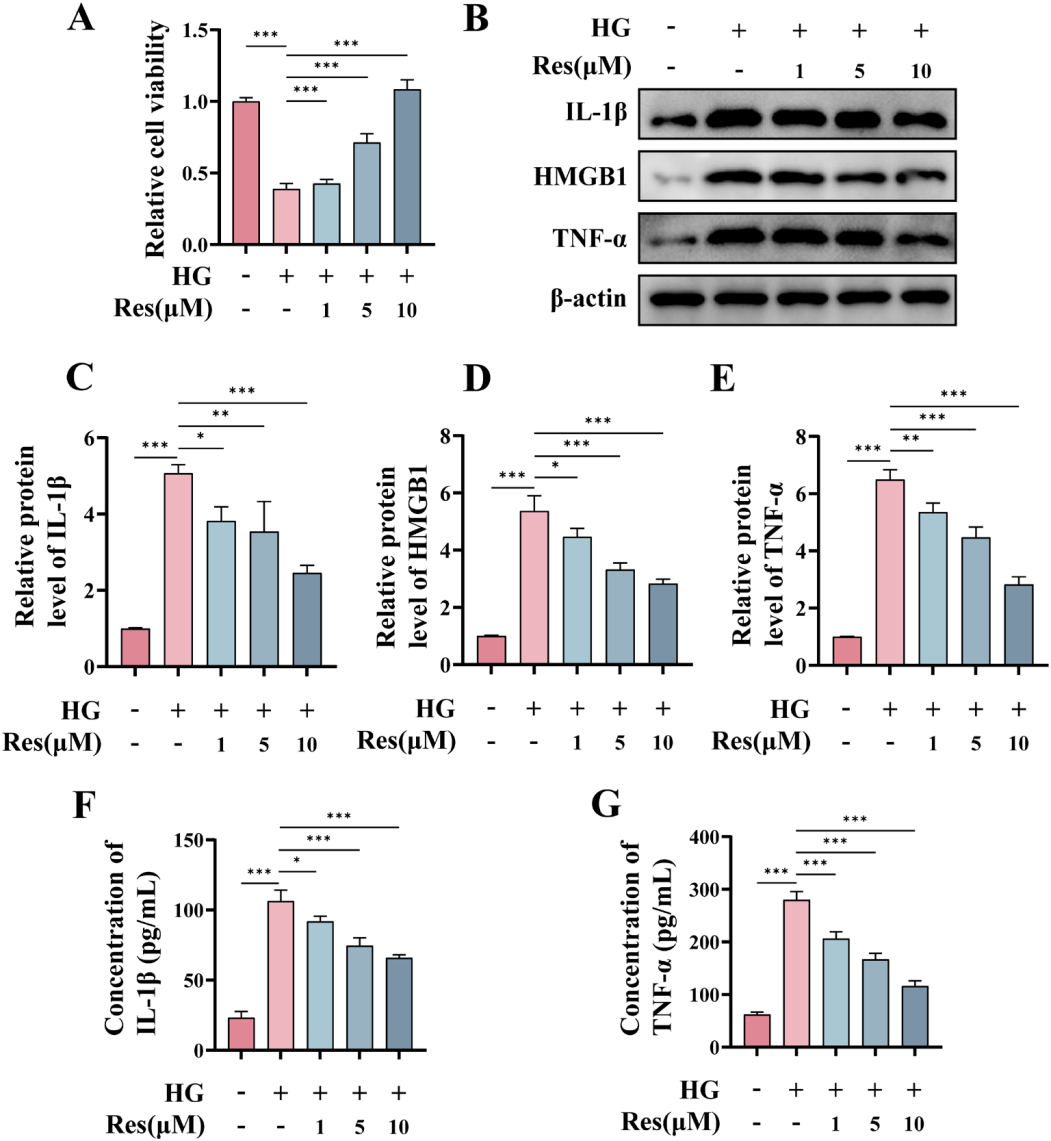
Res alleviates HG‐induced cellular inflammation in HUVECs. HUVECs were pretreated with or without Res (1, 5, or 10 μM) for 2 h, and then exposed to HG (30 μM) for 24 h. (A) Cell viability was detected by CCK‐8 assay. (B–E) The protein levels of IL‐1β, HMGB1 and TNF‐α were detected by Western blot. (F, G) Concentration of cytokine IL‐1β and TNF‐α were detected by ELISA. *p < 0.05; **p < 0.01; ***p < 0.001.

Endothelial cells play a key role in the wound healing process by promoting angiogenesis and regulating inflammatory responses. However, the local hyperglycemic condition of diabetic wounds exacerbates the cellular inflammatory response, thereby impeding the wound healing process (Geng et al. 2023). To examine the efficacy of Res in mitigating HG‐induced cellular inflammation, we pretreated HUVECs with different concentrations of Res for 2 h, followed by exposure to HG for 24 h. The results demonstrated a significant reduction in HUVEC viability after exposure to HG; however, pretreatment with Res (1, 5, 10 μM) effectively counteracted the HG‐induced decline in cell viability (Figure 1A). As illustrated in Figure 1B–E, the levels of pro‐inflammatory proteins, including interleukin 1 beta (IL‐1β), tumor necrosis factor alpha (TNF‐α), and high mobility group box 1 (HMGB1), were elevated following HG treatment. However, these alterations were mitigated in a dose‐dependent manner upon administration of Res (1, 5, 10 μM). Additionally, the cytokine concentrations of IL‐1β and TNF‐α were quantified using ELISA, and similar results were obtained (Figure 1F,G). These findings suggest that Res effectively attenuates HG‐induced cellular inflammation.

### 3.2 Res Inhibits HG‐Induced Senescence and Angiogenic Dysfunction in HUVECs

**FIGURE 2:**
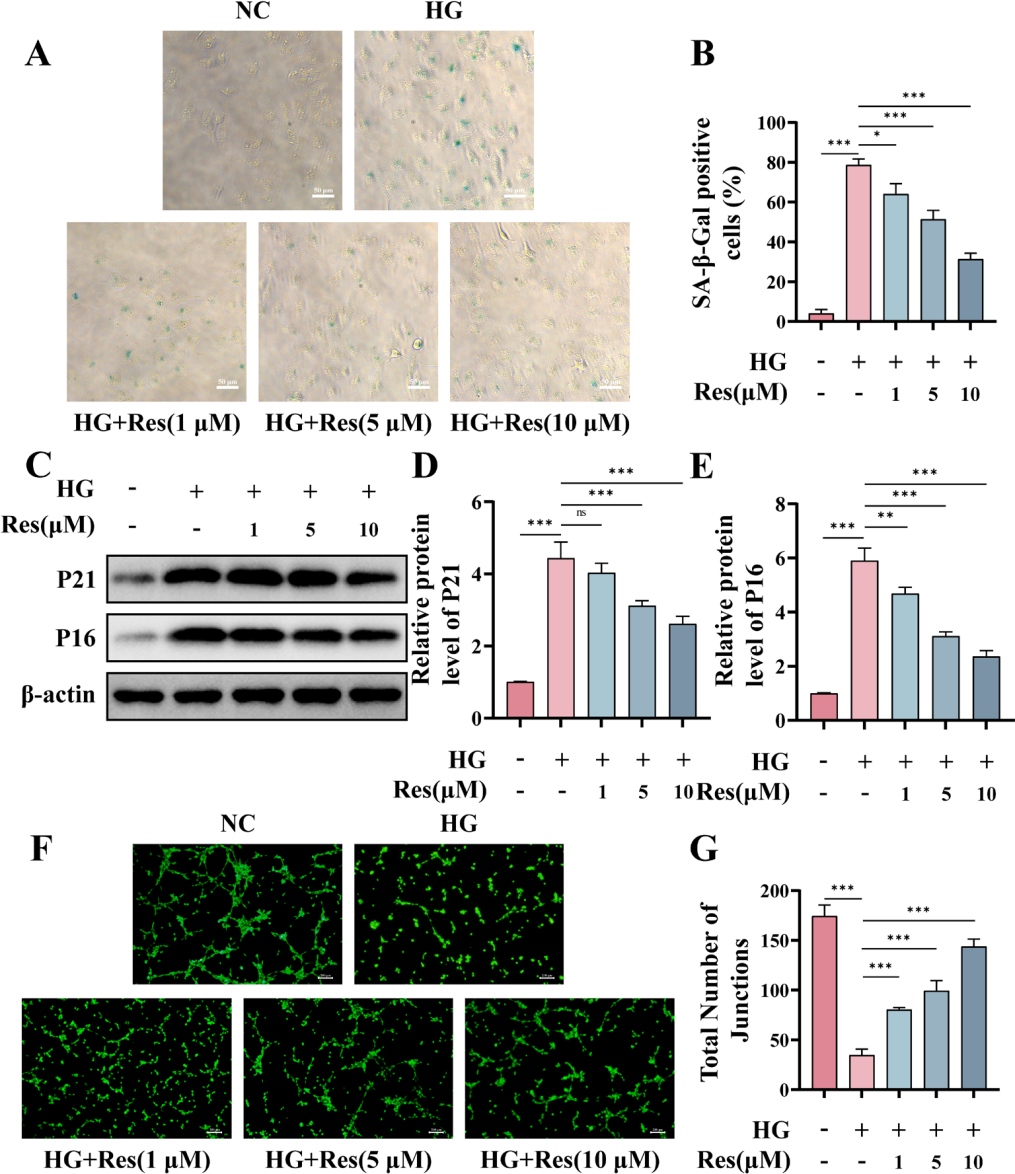
Res inhibits HG‐induced senescence and angiogenic dysfunction in HUVECs. HUVECs were pretreated with or without Res (1, 5, or 10 μM) for 2 h, and then exposed to HG (30 μM) for 24 h. (A, B) Representative image of SA‐β‐Gal staining of HUVECs. Scale bar = 50 μm. (C–E) The protein levels of P21 and P16 were detected by Western blot. (F, G) Tube formation assay in vitro and semi‐quantitative analysis of the total number of junctions. Scale bar = 200 μm. *p < 0.05; **p < 0.01; ***p < 0.001; ns, no significant difference.

Cellular senescence can be conceptualized as an accumulative process resulting from various forms of cellular damage, primarily characterized by the arrest of the cell cycle (Chen, Gan, et al. 2022). During wound healing, local hyperglycemic conditions induce senescence in endothelial cells, thereby impairing their angiogenic capacity (Huang et al. 2020). A marked elevation in the proportion of SA‐β‐Gal positive cells was observed in HUVECs 24 h after HG treatment. Conversely, pretreatment with Res led to a reduction in this proportion (Figure 2A,B). Subsequent analysis of the cyclin‐dependent kinase inhibitors P21 and P16 revealed a significant upregulation in their protein levels following HG treatment, whereas Res treatment resulted in a dose‐dependent decrease, suggesting that Res mitigates HG‐induced cellular senescence (Figure 2C–E). Furthermore, the tube formation assay in vitro demonstrated that Res effectively counteracted the HG‐induced suppression of tubular formation ability in HUVECs (Figure 2F,G).

### 3.3 Res Promotes the Healing Process of Wounds in a Diabetic Mouse Model

**FIGURE 3:**
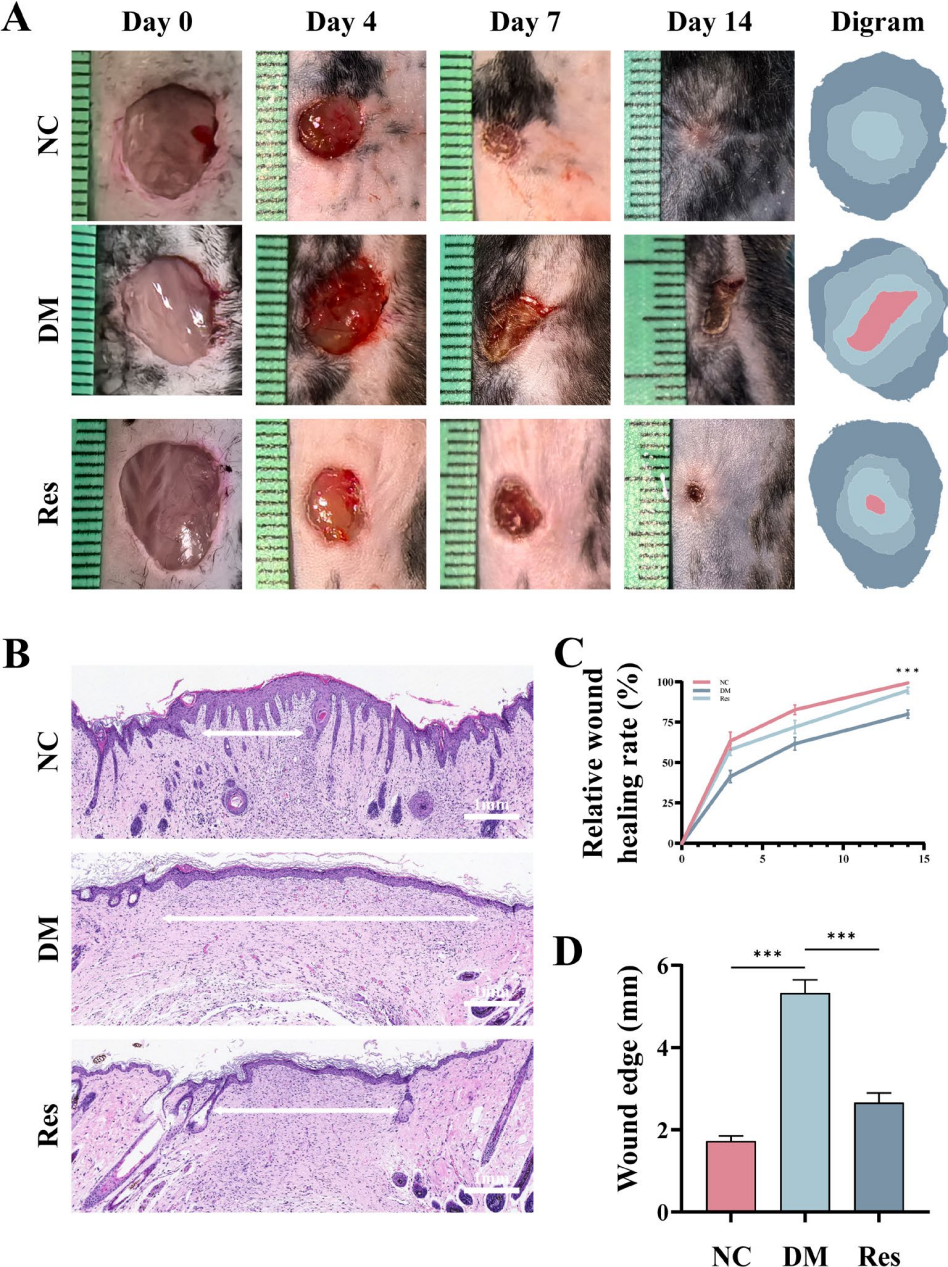
Res promotes the healing process of wounds in a diabetic mouse model. (A, C) Representative images and quantification of the wound healing process at the indicated time point (*n* = 6). (B, D) H&E staining of wound tissue on the 14th day (*n* = 6). Scale bar = 1 mm. ***p < 0.001.

The STZ‐induced wound model in diabetic mice was employed to assess the in vivo efficacy of Res in enhancing wound healing. Observations were systematically conducted on days 0, 4, 7, and 14. On day 14, tissue samples were harvested from euthanized mice for subsequent analyses. Our observations indicated a temporal trend in wound healing across all groups; however, the rate of wound healing in the DM group was markedly lower compared to the NC group. Treatment with Res significantly enhanced the wound healing process in diabetic mice (Figure 3A,C). Furthermore, H&E staining revealed a substantial improvement in skin epithelialization in the Res‐treated group relative to the untreated diabetic group (Figure 3B,D).

**FIGURE 4:**
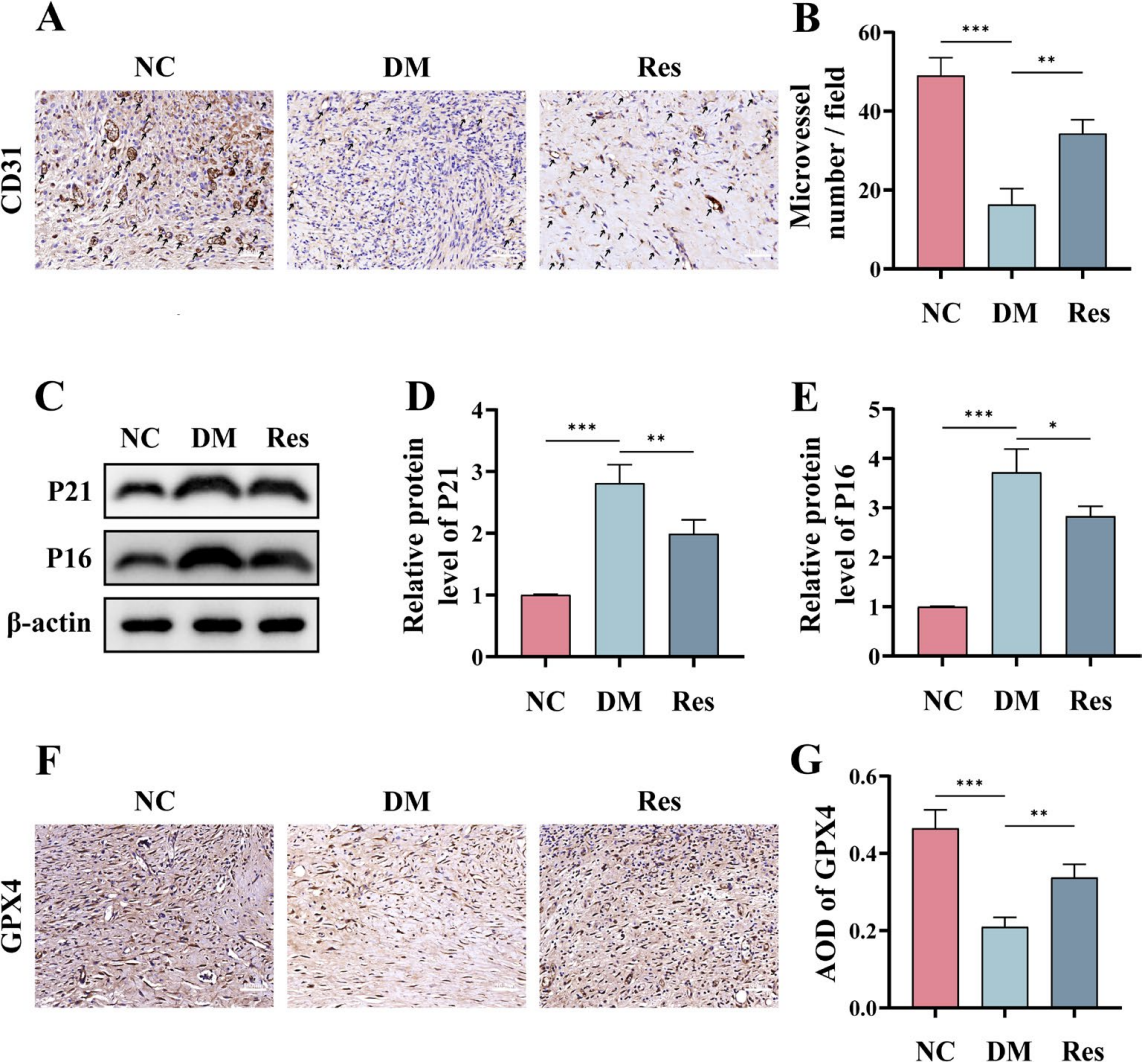
Potential mechanisms of Res in promoting diabetic wound healing. (A, B) Immunohistochemistry of CD31 in the wound tissue on day 14 (*n* = 6). Scale bar = 100 μm. (C–E) Western blot detection and semi‐quantitative analysis of P21 and P16 protein levels in wound tissue. (F, G) Immunohistochemistry and semi‐quantitative analysis of AOD for GPX4 in the wound tissue on day 14 (*n* = 6). Scale bar = 100 μm. *p < 0.05; **p < 0.01; ***p < 0.001.

Angiogenesis is crucial for the repair of diabetic wounds, and its impairment frequently results in delayed wound healing (Chang and Nguyen 2021). CD31 staining of tissue sections at 14 days revealed a diminished angiogenic capacity in mice induced by STZ. However, an increase in neovascularization was observed in the wound tissue of the Res group compared to the DM group, indicating that Res facilitates wound healing (Figure 4A,B). The protein levels of P21 and P16 in wound tissues across each group were quantified, revealing an upregulation of these proteins in the DM group, which implies a potential involvement of aging mechanisms in the impaired healing process of diabetic wounds. Notably, the Res group exhibited a significant reduction in P21 and P16 protein levels compared to the DM group, paralleling findings observed in HG‐induced HUVECs (Figure 4C–E). Subsequently, immunohistochemical staining was employed to evaluate the expression levels of GPX4 in each group (Figure 4F,G). A semiquantitative analysis of the average optical density (AOD) revealed a significant reduction in AOD in the DM group compared to the NC group; however, this reduction was notably mitigated by Res. These findings indicate that Res may facilitate the healing of diabetic wounds by inhibiting the ferroptosis pathway associated with diabetic wound pathology.

### 3.4 Res Attenuates HG‐Induced Ferroptosis in HUVECs

**FIGURE 5:**
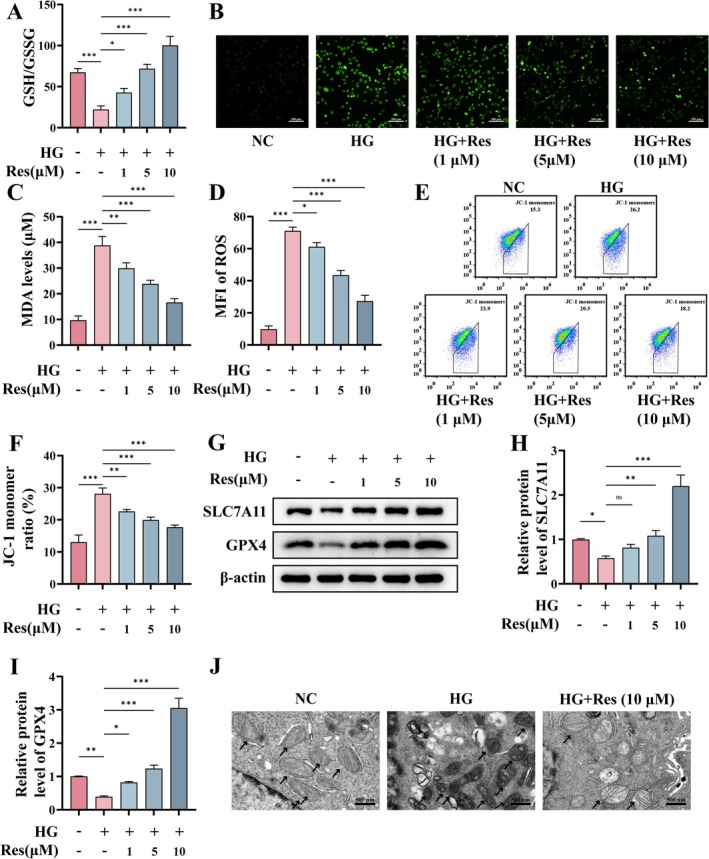
Res attenuates HG‐induced ferroptosis in HUVECs. HUVECs were pretreated with or without Res (1, 5, or 10 μM) for 2 h, and then stimulated with HG (30 μM) for 24 h. (A) The ratio of GSH to GSSG was quantified by the GSH and GSSG assay kit. (B, D) DCFH‐DA staining and MFI analysis of ROS. Scale bar = 100 μm. (C) MDA levels were detected by the lipid peroxidation kit. (E, F) Flow cytometric analysis of JC‐1. (G–I) The protein levels of SLC7A11 and GPX4 were detected by Western blot. (J) The transmission electron microscope images of mitochondrial morphology. Scale bar = 500 nm. *p < 0.05; **p < 0.01; ***p < 0.001; ns, no significant difference.

Building on in vivo and in vitro observations, we conducted a subsequent investigation using HG‐induced HUVECs to determine whether Res can mitigate endothelial cell ferroptosis precipitated by a hyperglycemic milieu. We quantified the concentrations of GSH and its oxidized form, GSSG, and discovered that HG treatment markedly diminished intracellular GSH levels. In contrast, Res demonstrated a dose‐dependent enhancement in GSH concentrations (Figure 5A). Subsequently, our findings indicated that HG exposure resulted in an increase in intracellular ROS levels. This effect was mitigated by Res (1, 5, 10 μM), demonstrating concentration‐dependent effects (Figure 5B,D). Subsequent assessments of intracellular lipid peroxidation showed that Res effectively reduced the HG‐induced elevation of MDA levels (Figure 5C). Mitochondrial lipid peroxidation is considered critical for initiating ferroptosis, leading to mitochondrial dysfunction and increased ROS production, which further promotes ferroptosis (Chen, Wang, et al. 2022). The JC‐1 probe was employed to assess the impact of Res on MMP induced by HG. As shown in Figure 5E,F, JC‐1 monomer levels increased in HUVECs following HG treatment, indicating a collapse of MMP as well as an increase in mitochondrial membrane permeability. Conversely, Res (1, 5, 10 μM) attenuated the changes in MMP induced by HG.

Subsequent analysis focused on the ferroptosis‐associated proteins GPX4 and SLC7A11. The findings indicated that HG led to a reduction in GPX4 and SLC7A11 levels; however, Res (1, 5, 10 μM) effectively counteracted the suppressive impact of HG on these protein levels (Figure 5G–I). Furthermore, transmission electron microscopy was used to examine mitochondrial morphology. The analysis revealed that mitochondria in HUVECs exposed to HG showed increased density and shrinkage, along with a reduction in cristae, which are characteristic of ferroptosis. In contrast, mitochondria treated with Res (10 μM) demonstrated a decrease in density and a near restoration of normal volume, although some mitochondrial cristae fractures persisted (Figure 5J). These findings indicate that Res mitigates HG‐induced ferroptosis in HUVECs. In conjunction with Figure 4F, the results indicate that Res possesses the capability to inhibit ferroptosis both in vivo and in vitro.

### 3.5 Res Reduces HG‐Induced Cellular Inflammation, Senescence, and Angiogenic Dysfunction Through the Inhibition of Ferroptosis

**FIGURE 6:**
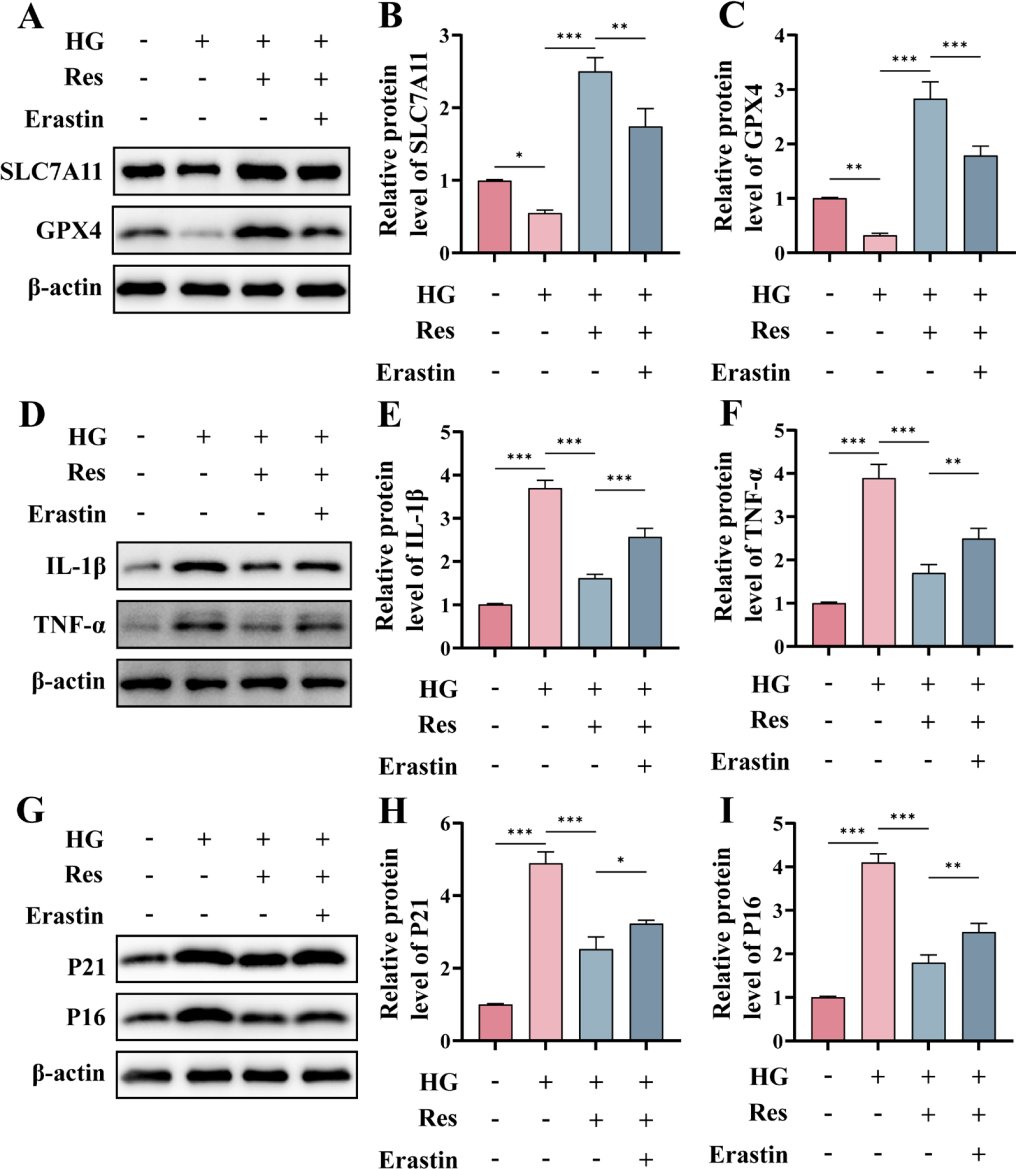
Res reduces HG‐induced cellular inflammation, senescence, and angiogenic dysfunction through the inhibition of ferroptosis. HUVECs were pretreated with or without Res (10 μM) for 2 h, and then HG (30 μM) or Erastin (5 μM) was added and cultured for 24 h. (A–C) The protein levels of SLC7A11 and GPX4 were assessed using Western blot. (D‐F) The protein levels of IL‐1β and TNF‐α. (G–I) The protein levels of P21 and P16. *p < 0.05; **p < 0.01; ***p < 0.001.

To confirm that Res facilitates angiogenesis by inhibiting HG‐induced ferroptosis in HUVECs, we employed the ferroptosis agonist Erastin to counteract the inhibitory effects of Res on ferroptosis. As shown in Figure 6A–C, Erastin treatment led to a loss of Res‐mediated enhancement of GPX4 and SLC7A11 protein levels. Additionally, the Erastin‐induced promotion of ferroptosis reinstated the protein levels of TNF‐α and IL‐1β, suggesting that Res reduces cellular inflammation by inhibiting ferroptosis (Figure 6D–F). Furthermore, Erastin antagonized the inhibitory effects of Res on the cyclin‐dependent kinase inhibitors P21 and P16 (Figure 6G–I). These findings indicate that Res mitigates HG‐induced cellular inflammation, senescence, and angiogenic dysfunction by inhibiting ferroptosis.

**FIGURE 7:**
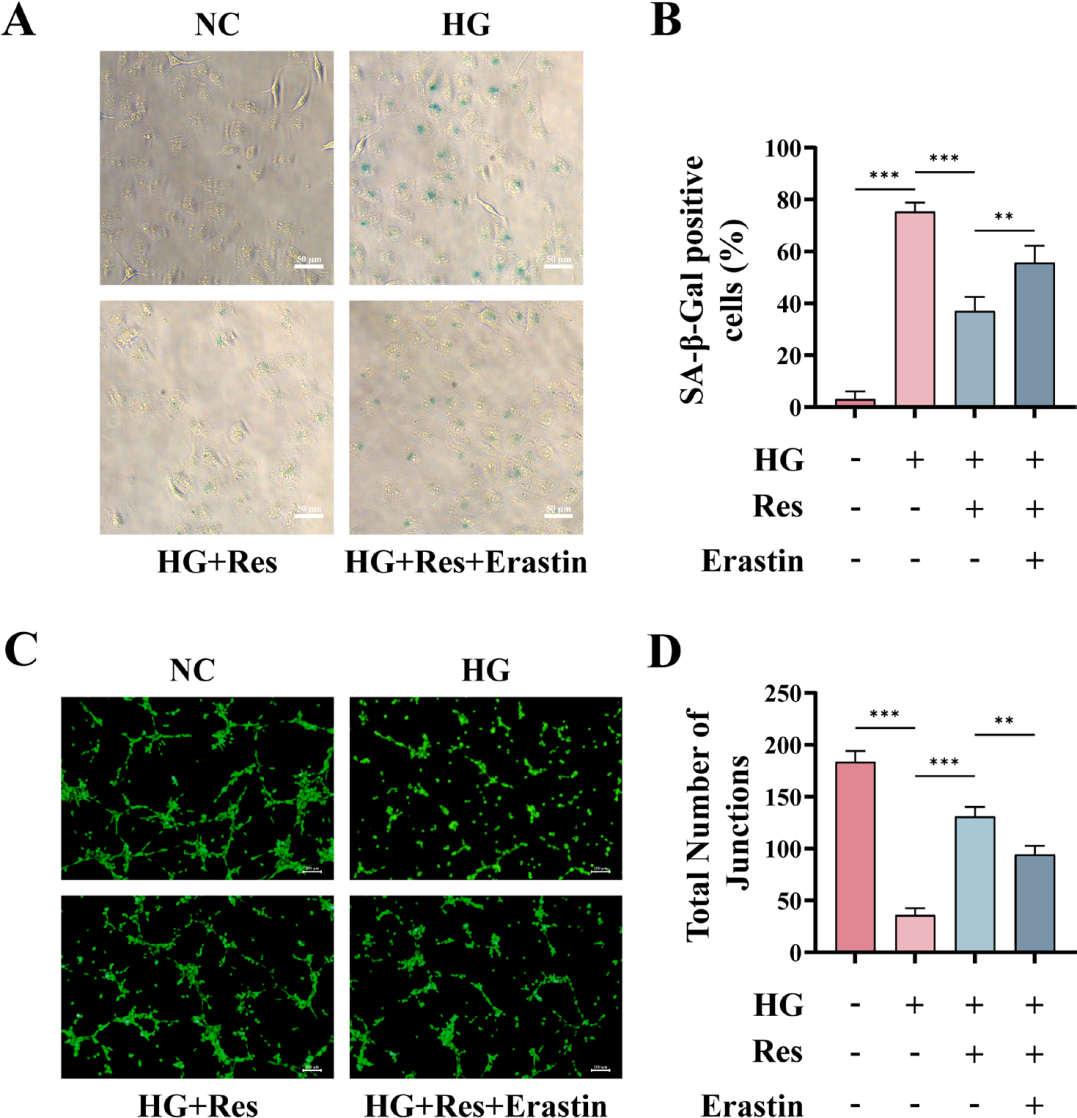
Res reduces HG‐induced senescence and angiogenic dysfunction through the inhibition of ferroptosis. HUVECs were pretreated with or without Res (10 μM) for 2 h, and then HG (30 μM) or Erastin (5 μM) was added and cultured for 24 h. (A, B) Representative image of SA‐β‐Gal staining of HUVECs. Scale bar = 50 μm. (C, D) Tube formation assay in vitro and semi‐quantitative analysis of the total number of junctions. Scale bar = 200 μm. **p < 0.01; ***p < 0.001.

Alongside this, Erastin increased the proportion of SA‐β‐Gal positive cells compared to the Res group (Figure 7A,B). These results indicated that Res resists HG‐induced senescence by inhibiting ferroptosis. Moreover, angiogenesis assays demonstrated that Erastin attenuated the pro‐angiogenic effects induced by Resin HUVECs under hyperglycemic conditions (Figure 7C,D).

### 3.6 Res Mitigates HG‐Induced Ferroptosis Through the Activation of the PI3K‐AKT‐Nrf2 Signaling Pathway

**FIGURE 8:**
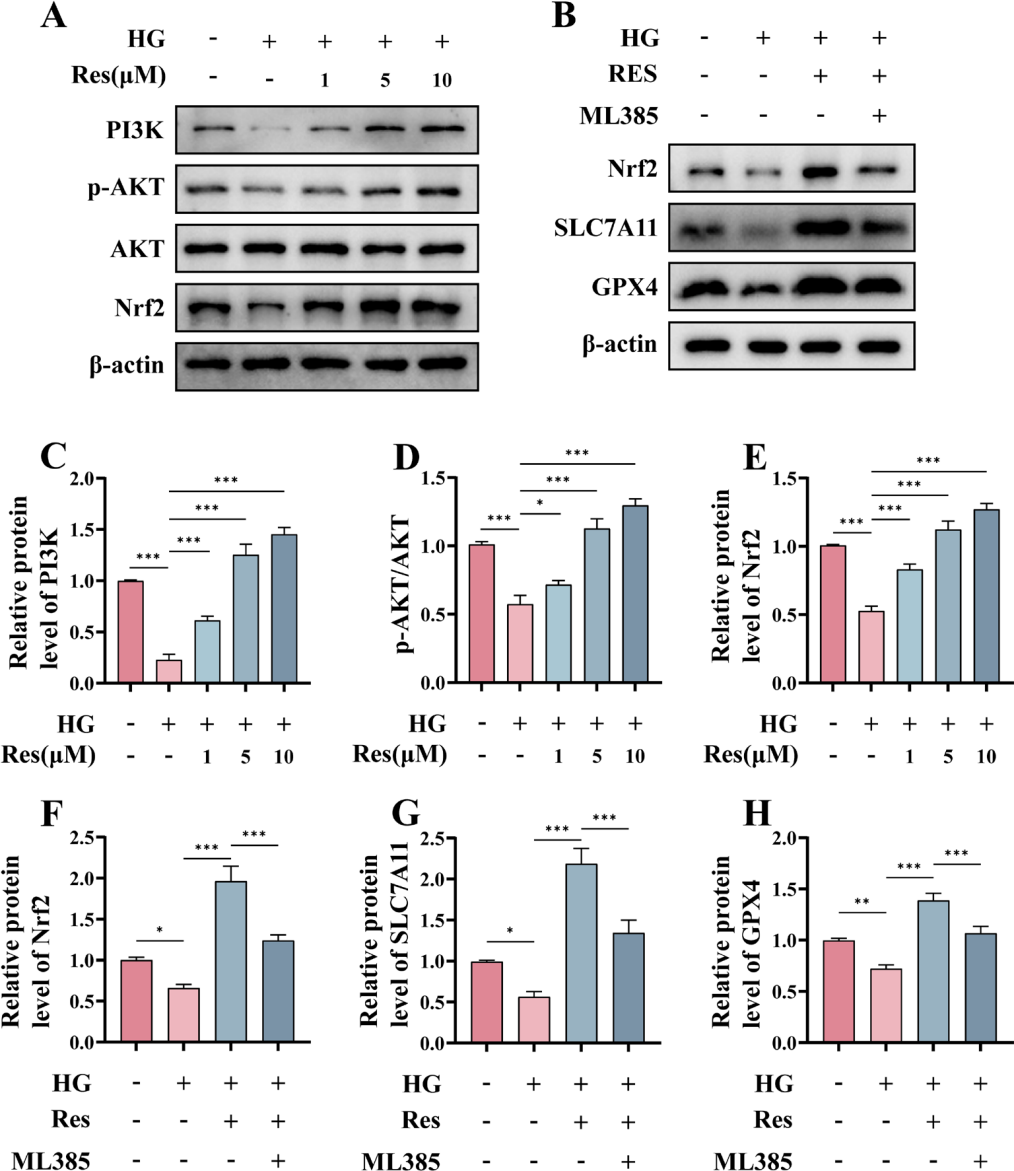
Res mitigates HG‐induced ferroptosis through the activation of the PI3K‐AKT‐Nrf2 signaling pathway. HUVECs were pretreated with or without Res (1, 5, 10 μM) for 2 h, and then HG (30 μM) was added and cultured for 24 h. (A, C, D, and E) The protein levels of PI3K, p‐AKT, AKT and Nrf2 were assessed using Western blot. HUVECs were pretreated with or without Res (10 μM) for 2 h, and then HG (30 μM) or ML385 (5 μM) was added and cultured for 24 h. (B, F, G, and H) The protein levels of Nrf2, SLC7A11 and GPX4. *p < 0.05; **p < 0.01; ***p < 0.001.

The activation of the phosphoinositide 3‐kinase (PI3K) leads to the phosphorylation of protein kinase B (AKT), subsequently activating the downstream nuclear factor erythroid 2‐related factor 2 (Nrf2). Upon activation, Nrf2 upregulates the expression of various antioxidant genes, such as *GPX4*, which exerts a protective role against ferroptosis (Chen, Dong, et al. 2024). Therefore, we assessed the protein levels of PI3K, p‐AKT, AKT, and Nrf2 (Figure 8A,C–E). HG treatment markedly diminished the protein levels of PI3K and the phosphorylation of AKT, subsequently leading to a reduction in downstream Nrf2 protein levels. Conversely, Res significantly counteracted this effect in a dose‐dependent manner. The Nrf2‐specific inhibitor ML385 was employed to further investigate whether Res inhibits ferroptosis in HUVECs through the PI3K‐AKT‐Nrf2 signaling pathway (Figure 8B,F–H). Consistent with expectations, the protein levels of Nrf2 were reduced in the ML385‐treated group compared to the Res‐treated group. Additionally, the ferroptosis‐associated proteins SLC7A11 and GPX4 also decreased. These findings indicate that Res mitigates HG‐induced ferroptosis in HUVECs via the PI3K‐AKT‐Nrf2 signaling pathway.

## 4 Discussion

HG, as an initial factor, not only promotes the pro‐inflammatory polarization of macrophages (Yu et al. 2019) but also results in impaired migration and proliferation of endothelial cells (Zhou et al. 2024) and fibroblast dysfunction (Liu, Liu, et al. 2022), thereby hindering the healing process of diabetic wounds. Furthermore, prolonged exposure to HG can induce the condensation, rearrangement, cleavage, and oxidation of the free amino groups in proteins, amino acids, nucleic acids, and other macromolecular substances, as well as the aldehyde groups in reducing sugars. This process leads to the formation of AGEs, which contribute to wound inflammation disorders, impair angiogenesis, disrupt extracellular matrix deposition, and consequently impede wound healing (Zhu et al. 2022). Additionally, the hyperglycemic microenvironment of diabetic wounds is capable of inducing endothelial cell senescence by elevating intracellular ROS levels, accumulating AGEs, and triggering cellular inflammatory responses. This further aggravates the microenvironment of diabetic wounds and eventually impedes wound healing (Berlanga‐Acosta et al. 2020). Res, a naturally occurring polyphenolic compound, has been shown to effectively promote wound healing (Khazaei, Alizadeh, et al. 2024; Khazaei, Rahmati, et al. 2024). For instance, Li et al. utilized microneedles to deliver Res locally, thereby exerting its antioxidant properties and facilitating the healing of diabetic wounds (Li et al. 2024). In animal studies, Maleki et al. demonstrated that the combination of niacinamide riboside and Res effectively prevented pyroptosis in diabetic wounds and enhanced wound healing (Hasan Maleki et al. 2024). Similarly, Ding et al. observed that Res reduced the secretion of pro‐inflammatory cytokines TNF‐α and IL‐1β by macrophages, increased the expression of Arg‐1 and CD206, and promoted macrophage polarization towards the M2 phenotype, thereby contributing to the healing of diabetic wounds (Ding, Yang, et al. 2022). In this study, we demonstrated that Res facilitates angiogenesis and accelerates wound healing by alleviating HG‐induced endothelial cell inflammation and senescence.

Iron‐dependent cell death, known as ferroptosis, is a distinct form of cell death characterized primarily by lipid peroxidation. It has been shown to have a significant association with the pathological processes involved in diabetic wound healing (Yu et al. 2024). Specifically, the hyperglycemic microenvironment in diabetic wounds leads to the excessive accumulation of ROS, subsequently resulting in lipid peroxidation in key cells (including endothelial cells, fibroblasts, and keratinocytes), and thereby triggering ferroptosis (Feng et al. 2022). Moreover, diabetic wounds are frequently associated with abnormal iron metabolism, characterized by iron overload or disordered iron distribution. As a pivotal driver of ferroptosis, excessive free iron catalyzes the production of substantial amounts of ROS via the Fenton reaction, further exacerbating lipid peroxidation and promoting cell death (Bi, Li, et al. 2023). In vitro studies have indicated that the HG environment can result in a decline in the expression of ferroptosis markers GPX4 and SLC7A11 in endothelial cells and fibroblasts, thereby inducing ferroptosis (Li et al. 2021). Additionally, ferroptosis may amplify the accumulation of ROS and lipid peroxidation products through a positive feedback mechanism, thereby impeding diabetic wound healing (Feng et al. 2022). Luo et al. demonstrated that HG can reduce the expression of GPX4 in endothelial cells through activation of the p53‐xCT‐GSH pathway, resulting in endothelial cell dysfunction. This process is mitigated by the application of ferroptosis inhibitors, suggesting that HG‐induced ferroptosis contributes to endothelial cell dysfunction (Luo et al. 2021). The endothelial cell dysfunction caused by ferroptosis is not only manifested as compromised angiogenesis but may also activate immune cells by releasing damage‐associated molecular patterns (such as HMGB1), intensifying local inflammatory responses and further exacerbating the wound healing process (Han et al. 2024).

Although current research on the relationship between Res and ferroptosis in the treatment of diabetic wounds remains limited, Res has demonstrated the ability to inhibit ferroptosis across various diseases. For example, Wang et al. reported that Res protects retinal Müller cells from ferroptosis in the early stages of diabetic retinopathy by activating the Nrf2‐GPX4‐PTGS2 pathway (Wang et al. 2024). Furthermore, in research investigating intestinal mucosal ischemia–reperfusion injury, Res has been found to ameliorate the injury by inhibiting ROS‐induced ferroptosis in intestinal mucosal cells through the activation of the SIRT3‐FoxO3a pathway. In a similar manner, Res has demonstrated cardioprotective effects by preventing DOX‐induced ferroptosis in cardiomyocytes (Chen, Sun, et al. 2024). In this study, elevated levels of ferroptosis were detected in the model group under both in vivo and in vitro conditions. Therefore, we propose the hypothesis that Res facilitates the healing of diabetic wounds by inhibiting ferroptosis. In vitro studies demonstrated that HG conditions not only result in the depletion of GSH and an increase in ROS but also cause the collapse of MMP, ultimately leading to ferroptosis (Figure 5). However, pretreatment with Res ameliorated these effects. Furthermore, the application of ferroptosis agonists restored the aforementioned biological effects of resveratrol, demonstrating that Res mitigates the inflammatory response and decelerates the aging process of endothelial cells by inhibiting ferroptosis.

Further investigation into the mechanism by which Res inhibits ferroptosis revealed that Res effectively suppresses ferroptosis via activation of the PI3K‐AKT‐Nrf2 signaling pathway, thereby protecting HUVECs against inflammation and senescence induced by high glucose (HG) conditions. The activation of the PI3K‐AKT signaling pathway elevates GPX4 protein levels via Nrf2 and directly influences the activity of the cystine/glutamate antiporter system (System Xc^−^), including SLC7A11, thereby leading to enhanced intracellular cystine transport (Lim et al. 2019). Consequently, this promotes GSH synthesis, aligning with the experimental results observed in our study (Figure 5A,G). These findings provide novel insights into the mechanisms underlying nonhealing diabetic wounds and highlight the therapeutic potential of Res for their treatment.

## 5 Limitations and Future Perspectives

While this study provides valuable insights into the therapeutic potential of Res for diabetic wound healing, several limitations should be acknowledged. First, although the STZ‐induced diabetic mouse model is well‐established and widely utilized in preclinical research, it may not fully recapitulate the chronic hyperglycemic microenvironment and multifactorial pathogenesis characteristic of human diabetic wounds. Nonetheless, due to its relatively lower individual variability compared to diabetic transgenic mice, the STZ‐induced model remains a commonly preferred choice in contemporary diabetic wound healing research. Consequently, the in vivo experiments conducted using the STZ model in this study can, to a considerable extent, reflect the promoting effect of Res on diabetic wound healing. Second, our use of HG‐induced endothelial cells as the primary research model may not fully encompass the complex cellular interactions that occur in actual diabetic wounds, particularly the critical crosstalk among fibroblasts, keratinocytes, and immune cells that orchestrates the healing process. Nevertheless, this study was initiated based on the in vivo observation that Res could alleviate ferroptosis in diabetic wounds. Subsequent experiments were systematically designed and conducted to further explore its underlying mechanisms. In these follow‐up studies, we focused specifically on endothelial cells for mechanistic investigation, and the results obtained demonstrate considerable reliability and reference value for future research.

To address these limitations in future studies, several approaches could be implemented. More sophisticated in vitro systems, such as 3D coculture platforms incorporating multiple relevant cell types or advanced organ‐on‐a‐chip technologies, could better mimic the diabetic wound microenvironment. For in vivo investigations, future clinical translation studies could build upon more sophisticated animal models incorporating longitudinal assessment protocols and multi‐omics approaches. This would enable a more comprehensive evaluation of Res's therapeutic efficacy in diabetic wound healing through integrated high‐throughput sequencing techniques (e.g., single‐cell RNA sequencing and spatial transcriptomics) combined with functional wound healing parameters.

In summary, our current findings establish an important mechanistic foundation for understanding resveratrol's therapeutic effects. The results should be interpreted in light of these model constraints, while the identified limitations provide clear directions for more comprehensive future investigations that could bridge the gap between these preclinical findings and potential clinical applications.

## 6 Conclusion

In conclusion, our study demonstrates that Res exhibits significant therapeutic potential in promoting diabetic wound healing via multiple mechanisms. Specifically, Res effectively suppresses the inflammatory response and mitigates cellular senescence in endothelial cells within the diabetic wound microenvironment. These effects collectively enhance angiogenesis and expedite wound closure under diabetic conditions. Notably, our mechanistic studies reveal that the therapeutic effects of Res are predominantly mediated by its inhibition of ferroptosis through the activation of the PI3K‐AKT‐Nrf2 signaling pathway. Collectively, these findings not only elucidate the molecular mechanisms underlying Res‐mediated wound healing but also underscore its promise as a potential therapeutic agent for managing diabetic wounds.

## Author Contributions

**Yujie Pan:** data curation (lead), formal analysis (equal), investigation (equal), methodology (lead), software (lead), writing – original draft (lead). **Mingyan Xia:** conceptualization (lead), data curation (supporting), formal analysis (supporting), methodology (supporting), writing – original draft (supporting), writing – review and editing (lead). **Jin Luo:** investigation (supporting), methodology (supporting), software (lead). **Shuai Lu:** funding acquisition (lead), project administration (lead), supervision (lead), validation (lead).

## Ethics Statement

All animal procedures were approved by the Animal Care and Welfare Committee of Guizhou Medical University (Approval Number: 2400667) on January 13, 2024. The animal procedures adhered to Chinese national standards for laboratory animal welfare. Measures were implemented to minimize animal suffering, including the administration of anesthesia and adherence to humane euthanasia protocols.

## Conflicts of Interest

The authors declare no conflicts of interest.

## Data Availability

The data that support the findings of this study are available on request from the corresponding author.
